# BH3-Only Proteins Noxa and Puma Are Key Regulators of Induced Apoptosis

**DOI:** 10.3390/life12020256

**Published:** 2022-02-09

**Authors:** Rabih Roufayel, Khaled Younes, Ahmed Al-Sabi, Nimer Murshid

**Affiliations:** College of Engineering and Technology, American University of the Middle East, Kuwait; khaled.younes@aum.edu.kw (K.Y.); ahmed.al-sabi@aum.edu.kw (A.A.-S.); Nimer.Murshid@aum.edu.kw (N.M.)

**Keywords:** Noxa, Puma, Bcl-2 proteins, p53, heat-shock, caspase, ion channels, apoptosis

## Abstract

Apoptosis is an evolutionarily conserved and tightly regulated cell death pathway. Physiological cell death is important for maintaining homeostasis and optimal biological conditions by continuous elimination of undesired or superfluous cells. The BH3-only pro-apoptotic members are strong inducers of apoptosis. The pro-apoptotic BH3-only protein Noxa activates multiple death pathways by inhibiting the anti-apoptotic Bcl-2 family protein, Mcl-1, and other protein members leading to Bax and Bak activation and MOMP. On the other hand, Puma is induced by p53-dependent and p53-independent apoptotic stimuli in several cancer cell lines. Moreover, this protein is involved in several physiological and pathological processes, such as immunity, cancer, and neurodegenerative diseases. Future heat shock research could disclose the effect of hyperthermia on both Noxa and BH3-only proteins. This suggests post-transcriptional mechanisms controlling the translation of both Puma and Noxa mRNA in heat-shocked cells. This study was also the chance to recapitulate the different reactional mechanisms investigated for caspases.

## 1. Introduction

Apoptosis is a regulated form of genetically programmed cell death that has significant roles in development, tissue homeostasis, and in response to environmental stresses. Regulated cell death was first described as shrinkage necrosis, due to morphological observations, and later renamed apoptosis. The term apoptosis is derived from the Greek prefix “apo-”, which means “to separate”, and the suffix “-ptosis”, which means ‘to fall off’ [1,2,3]. During the first phase of apoptosis, termed the condensation phase, the cell separates from neighboring cells, the cytoplasm and nucleus become condensed, and nuclear DNA is fragmented [3]. The endoplasmic reticulum (ER), Golgi apparatus and mitochondria become disrupted [4]. This will intrigue the occurrence of regulated proteolysis [5,6]. The cell is then fragmented into apoptotic bodies [3,4]. The second stage of apoptosis involves phagocytosis and the destruction of apoptotic bodies [3,7]. Mutations in genes regulating apoptosis have been implicated in several human diseases including cancer [8,9,10,11,12].

Autophagy and apoptosis are key biochemical mechanisms that keep organismal and cellular homeostasis in check. Autophagy preserves cellular homeostasis by recycling selected intracellular organelles and chemicals, whereas apoptosis fulfills its job by demolishing damaged or undesired cells. Autophagy is a cell’s process of degrading its own cytoplasmic material. In other words, the cell’s interior contents deteriorate. Autophagy is derived from the words “auto-” (self) and “phagy” (eating). As a result, autophagy causes the cell to practically consume itself. This digestion takes place in the lysosome, which is commonly referred to as a cell’s stomach [13]. This mechanism, which permits intracellular organelles and proteins to be recycled, assists cells by eliminating damaged or undesirable organelles and macromolecules and providing energy and building materials for cellular operations and de novo biosynthesis [14,15].

Several molecular nodes of crosstalk have recently been discovered to connect autophagy with apoptosis, allowing for the coordinated regulation of degradation by both processes. Autophagy and apoptosis are normally tumor-suppressing mechanisms [16]. Autophagy fulfills this duty by facilitating the breakdown of oncogenic chemicals, inhibiting cancer formation, whereas apoptosis stops cancer cells from surviving. As a result, cancer can result from improper or insufficient amounts of autophagy or apoptosis [17,18].

The B-cell lymphoma-2 (Bcl-2) family proteins have a crucial role in controlling the apoptotic pathway [19,20,21,22,23]. The pro-apoptotic homology (BH), BH3-only protein, and Phorbol-12-myristate-13-acetate-induced protein 1 (Noxa), activates multiple death pathways, which can be achieved by inhibiting the anti-apoptotic Bcl-2 family protein myeloid cell leukemia-1 (Mcl-1), and other protein members. This ultimately activates Bcl-2-associated X protein (Bax), Bcl-2 homologous antagonist killer (Bak), and major outer mitochondrial membrane (OMM) proteins [19,24,25]. However, BH3-only protein, p53 upregulated modulator of apoptosis (Puma) is induced by p53-dependent and p53-independent apoptotic stimuli in several cancer cell lines. These family proteins are involved in many physiological and pathological processes [26,27], including the immune response [28,29,30], cancer [31,32,33,34], and neurodegenerative diseases [12,35,36,37].

The importance of the BH3-only proteins Noxa and Puma in apoptosis is summarized in this study. Herein, we start with the discussion of the Bcl-2 proteins and mitochondrial regulation, followed by the regulations of apoptosis including elucidation of the caspases reaction mechanism. In the fourth and fifth sections, we include the discussion of the BH3-only protein Noxa and Puma, respectively. Then, the vital role of ion channels in regulated cell death is also reviewed. The last section of this review discusses the heat shock response to Puma and Noxa protein expression in vitro.

## 2. Bcl-2 Proteins and the Regulation of Mitochondrial Outer Membrane Permeability

**Figure 1:**
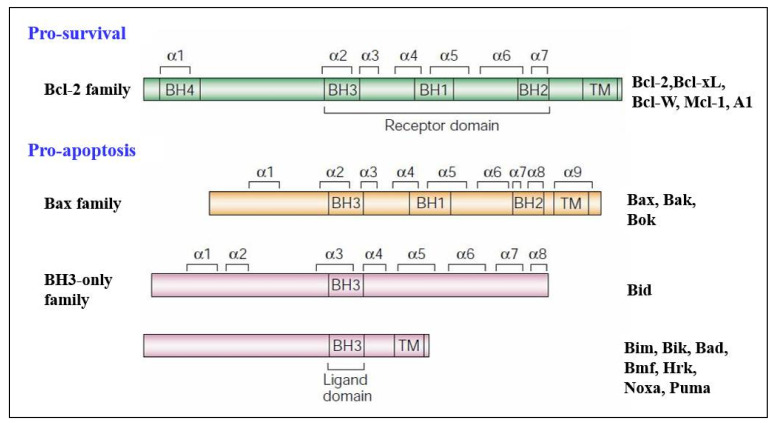
Structural comparison of Bcl-2 family proteins. Pro-survival proteins including the Bcl-2 family (Bcl-2, Bcl-xL, Bcl-W, Mcl-1, and A1) and the pro-apoptosis proteins: Bax family (Bax, Bak, and Bok) and BH3-only family (Bid, Bim, Bik, Bad, Bmf, Hrk, Noxa, and Puma). (Adapted with permission from Cory and Adams ref. [41]. Copyright 2001 Elsevier).

Molecular insights into apoptosis first emerged during the 1980s and 1990s from a convergence of mammalian cancer cytogenetics and genetic studies on developmentally programmed cell death in *Caenorhabditis* elegans [38]. The previously unknown gene Bcl-2 was identified from the breakpoint region of a recurrent chromosomal translocation (18q21) in human follicular lymphoma [7,39]. The Bcl-2 family of proteins are central regulators of stress-induced apoptosis as they control diverse survival and death signals that are generated inside and outside the cell [40,41]. Bcl-2 proteins are found in all cells and consist of 12 distinct members, many of which are expressed as alternate isoforms. Structurally, they all contain at least one of the conserved sequence motifs called Bcl-2 BH domains. This family is functionally subdivided into two classes based on their activity and number of BH domains: anti-apoptotic and pro-apoptotic proteins including the BH3-only members [19,41,42] (Figure 1). The mutual interaction between pro-apoptotic and anti-apoptotic members establishes the threshold that determines whether a cell should survive or die [43]. In a sense, they act as checkpoints through which survival and death signals must pass to elicit the cell’s fate as they control stress-induced cell death [44]. As shown in Figure 1, members of the Bcl-2 family can be divided into two categories. The first category contains pro-survival members and includes Bcl-2, Bcl-xL, Bcl-W, Mcl-1, and A1. These proteins contain multiple Bcl-2 homology (BH) domains (BH1-4), though certain members, such as Mcl-1, lack the N-terminal BH4 domain. Additionally, most pro-survival Bcl-2 family members, such as Mcl-1, contain a transmembrane (TM) domain that targets these members to specific intracellular compartments. The second category contains pro-apoptotic members, which can be further subdivided into the Bax family and the BH3-only family. The Bax family consists of Bax, Bak and Box, all of which are responsible for forming oligomeric pores in the outer mitochondrial membrane. This results in the release of cytochrome c and the activation of apoptosis. These proteins contain multiple BH domains (BH1-3) and a TM domain that guides them to the outer mitochondrial membrane. The BH3-only family consists of BH3 interacting-domain death agonist (Bid), Bcl-2-like protein 11 (Bim), Bcl-2-interacting killer (Bik), Bcl-2-associated death promoter (Bad), Bcl-2-modifying factor (Bmf), activator of apoptosis 3ara-kiri (Hrk), Noxa, and Puma. These proteins are responsible for activating Bax, Bak, and Bok upon receiving stress signals, though the exact mechanism is currently being investigated. All members of the BH3-only subfamily of Bcl-2 family proteins contain only a single BH domain (BH3), and most members, such as Noxa, also contain a TM domain responsible for targeting these proteins to specific intracellular compartments.

The anti-apoptotic Bcl-2 members, including Bcl-2 itself, Bcl-xL, Mcl-1, Bcl-W, and A1/BFL-1 share four conserved BH domains of structural homology. These proteins prevent cell death against diverse cytotoxic signals, both physiological and imposed. This maintains the integrity of the ER, mitochondrial, and nuclear membranes, thus protecting cells from apoptosis [45,46]. The proximity of BH 1, 2, and 3 form a hydrophobic pocket that operates as a receptor for the BH3 domain of the pro-apoptotic BH3-only members. The anti-apoptotic Bcl-2 proteins work together to inhibit the release of cytochrome c from the mitochondria by preventing the activation of the pro-apoptotic members Bax and Bak in the OMM [44]. The anti-apoptotic proteins’ normal function is to prevent inappropriate cell death and therefore requires the neutralization of the pro-apoptotic proteins by the anti-apoptotic members [47].

Just as the anti-apoptotic Bcl-2 proteins promote tumourigenesis when deregulated, the pro-apoptotic members function as tumor suppressors. The pro-apoptotic Bcl-2 members are divided into multi-domain effector proteins, such as Bax, Bak, and the less well-known Bok, as well as the large subgroup of BH3-only proteins, all of which trigger or sensitize the cell to apoptosis [48,49,50]. The pro-apoptotic effector proteins Bax, Bak, and Bok possess three BH domains and adopt similar globular structures: a helical bundle surrounding a central hydrophobic core helix [44,51,52]. This groove constitutes a crucial surface for interactions with the BH3 domain of pro-apoptotic members of the Bcl-2 family [38]. These interactions primarily occur on intracellular membranes, such as that of the OMM, where many of the Bcl-2 family members are directed by their carboxy-terminal hydrophobic TM domain [53]. Unlike the anti-apoptotic members, the active conformation of Bax/Bak damages rather than protects the OMM of stressed cells. This yields the formation of pores leading to membrane dysfunction [54]. The most studied pro-apoptotic proteins Bax and Bak exist in an inactive monomeric state in healthy cells. In stressed cells, they are major inducers of mitochondrial outer membrane permeabilization (MOMP) [55].

The BH3-only proteins form the largest subgroup of Bcl-2 proteins and include Bid, Bad, Bim, Bik, Noxa, Puma, Hrk, BNIP1/2/3, and Bmf, all of which have only the BH3 domain in common [52,56]. The pro-apoptotic BH3-only proteins act as sensors of diverse cytotoxic stress signals that act upstream of the anti-apoptotic members, inhibiting their ability to block Bax/Bak activation [38,47]. This occurs through interactions between the pro-apoptotic and anti-apoptotic proteins on the outer mitochondrial membrane.

**Figure 2:**
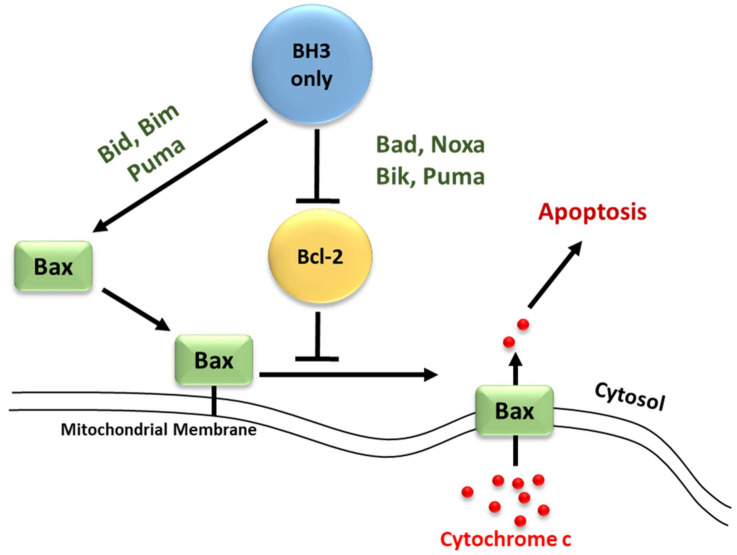
Direct and indirect activation of the intrinsic apoptotic pathway. Apoptosis is initiated by the release of cytochrome c from the mitochondria inter-membrane space to the cytosol via channels formed by the oligomerization of Bak or Bax in the OMM.

Recent studies have focused on the effects of the Bcl-2 family members on MOMP. Although over a decade has passed since the discovery of the BH3-only protein Puma, the question of how this protein activates Bax/Bak, consequently leading to MOMP, remains unresolved. At present, two mechanisms have been proposed concerning the relationship of the Bcl-2 protein family members employing direct or indirect activation of the mitochondrial apoptotic pathway, shown in Figure 2 [44,47,54]. Two models are explaining Bak/Bax activation through the interaction of the Bcl-2 protein family members: the direct activation model and the indirect activation model.

In the indirect model, Bax/Bak activation occurs only when the BH3-only proteins bind to and inhibit all anti-apoptotic Bcl-2 proteins [57]. This occurs through interactions between the amphipathic α-helix of the BH3 domain on the pro-apoptotic BH3-only members and the hydrophobic pocket of the BH1, 2, and 3 domains present on the target anti-apoptotic members, inactivating them and liberating the pro-apoptotic Bax/Bak [43,44,58]. Some of the BH3-only members have varying affinities for different anti-apoptotic proteins, whereas others can interact with all members [56,59]. Recent structural studies have shown a certain level of plasticity among the BH3 binding groove of the anti-apoptotic members, which probably contributes to their ability to associate with multiple BH3 domains [6]. Bim, Bid, and Puma bind all anti-apoptotic members with high affinity and thus are potent inducers of apoptosis, while Bad and Noxa which target only a few of the anti-apoptotic relatives are weak inducers [60]. The differential ability of certain BH3-only proteins to induce apoptosis when overexpressed can be explained by this differential specificity for anti-apoptotic members [61]. It is proposed that efficient apoptosis requires the neutralization of all anti-apoptotic members within a given cell [60,62]. Despite this, co-expression of certain members such as Bad and Noxa, with opposite specificity, target all the anti-apoptotic members allowing the activation and oligomerization of Bax/Bak in the OMM. The BH3-only proteins; however, are latent killers that require activation by distinct cytotoxic stimuli. This shift in Bax/Bak to their active conformation leads to MOMP and ultimately apoptosis of the cell [61,63].

Conversely, in the direct model, BH3-only proteins can directly activate the pro-apoptotic effectors Bax/Bak in the absence of the anti-apoptotic members. It is suggested that certain BH3-only members (Bid, Bim, and Puma) are known as activators, capable of interacting directly with Bax/Bak to induce conformational changes that lead to their activation and oligomerization [64]. Other BH3-only members (Bad, Noxa, Bik, and Puma) are classified as sensitizers [52]. These sensitizers act to sequester the anti-apoptotic members (Bcl-2, Bcl-xL, and Mcl-1) and thereby inhibit their ability to suppress Bax/Bak activation [65].

**Figure 3:**
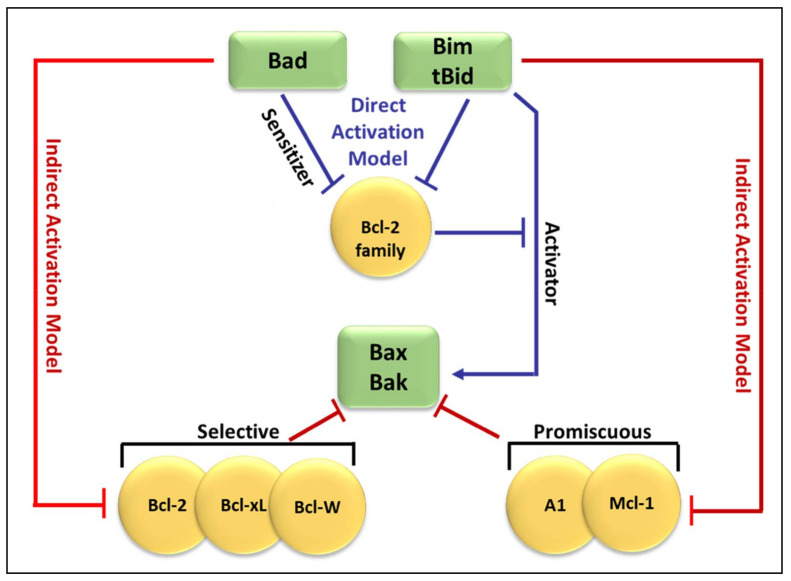
Comparison of the indirect and direct model of Bax and Bak activation. In the direct activation model (blue arrows), Bim and tBid act as “activators” by binding to Bak and Bax directly to induce pore formation. The remaining BH3-only proteins (Bid) bind to the Bcl-2-like proteins, releasing bound Bim and tBid which then bind directly to Bak and Bax. The indirect activation model (represented by red arrows from both sides), includes the engagement of anti-apoptotic Bcl-2-like proteins which then leads to the liberation of Bak and Bax.

Figure 3 compares both the indirect and direct models of Bax and Bak activation [47]. As shown in Figure 3, in the direct model (blue arrows), Bim and tBid act as “activators” by binding to Bak and Bax directly to induce pore formation [59]. The remaining BH3-only proteins act as “sensitizers” and bind to the anti-apoptotic Bcl-2-like proteins, releasing bound Bim and tBid and allowing them to directly activate Bak and Bax [57,66]. Additionally, some BH3-only proteins can only bind to specific anti-apoptotic Bcl-2 family proteins (selective) while others can bind to all anti-apoptotic Bcl-2 family proteins (promiscuous). This ability of BH3-only proteins to bind and neutralize certainly, or all, anti-apoptotic proteins is based on their BH3 domains [67].

By freeing up Bax/Bak, the sensitizers allow their activator relatives to bind and activate Bax/Bak [47,61]. In this model, Bid, Bim, and Puma are strong inducers of apoptosis both because of high affinities to all of the anti-apoptotic Bcl-2 members and due to their ability to bypass the anti-apoptotic inhibition step and directly activate Bax/Bak. Although two alternate models exist to describe the activation of Bax/Bak in the OMM, genetic and biochemical evidences support the idea that both the direct and indirect models apply in many circumstances. Thus, a unified model that operates within a tripartite network of interactions between the three subgroups of the Bcl-2 family of proteins [38]. The dominant pathway in this model varies depending on the biological state of the cells, such as non-transformed cells versus tumor cells or according to cytotoxic and cell death stimuli, and ultimately determines the fate of the cell [38]. Huang and co-workers demonstrated the “Membrane-mediated Permissive” model. They found that the BH3-only proteins activated Bax/Bak indirectly by neutralizing the anti-apoptotic Bcl-2 proteins. This ultimately activates the mitochondrial outer membrane and initiates apoptosis [25,63].

## 3. Regulation of Apoptosis

### 3.1. Regulators of Cell Death: Caspases

Caspases (Cysteinyl aspartate-specific proteases) are a family of proteins activated by a variety of stimuli representing a vital step for the induction of apoptosis. These proteases initiate and control the cellular death pathway by cleaving a diverse set of cellular proteins [68]. As previously discussed, chromatin condensation, nuclear DNA fragmentation, cell shrinkage, and membrane blebbing are predominantly due to caspase-dependent cleavage of key substrates such as; structural and cytoskeletal proteins, cell cycle machinery components, and proteins such as poly (ADP-ribose) polymerase (PARP) involved in genomic stability [4,43,69,70,71]. Two distinct caspase-dependent signaling pathways can induce apoptosis, the extrinsic pathway, and the intrinsic pathway [18,43]. Direct recruitment and activation of initiator caspases via the extracellular pathway are accomplished through a cytoplasmic domain on the tumor necrosis factor (TNF) family of death receptors [18,72,73]. The intrinsic apoptotic pathway is activated in response to intracellular stress and is mediated by the release of pro-apoptotic factors from the mitochondria [72]. Caspases are subdivided into two groups, the executioner caspases, which are responsible for dismantling the cells, and initiator caspases, which activate the executioner caspases by proteolytic processing. Caspases contain a pro-domain sequence at the N-terminal end followed by two subunits; p20 and p10 that comprise the catalytic core of the caspase [74,75]. For initiator caspases, the long N-terminal pro-domain promotes self-association and binding, forming a scaffolding system or activating adaptor proteins. All caspases are synthesized as inactive zymogens, called procaspases, and undergo activation when subjected to apoptotic stimuli resulting in induced dimerization or catalytic cleavage separating the p20 and p10 subunits [43,76]. Caspase-9, on the other hand, becomes activated by association with the apoptosome. In the presence of dATP and cytochrome c released from the inner mitochondrial membrane space, an allosteric change occurs in the tetrameric apoptotic protease activating factor 1 (Apaf1) forming the apoptosome [77,78].

### 3.2. Regulators of Cell Death: Bcl-2 Family Proteins

**Figure 4:**
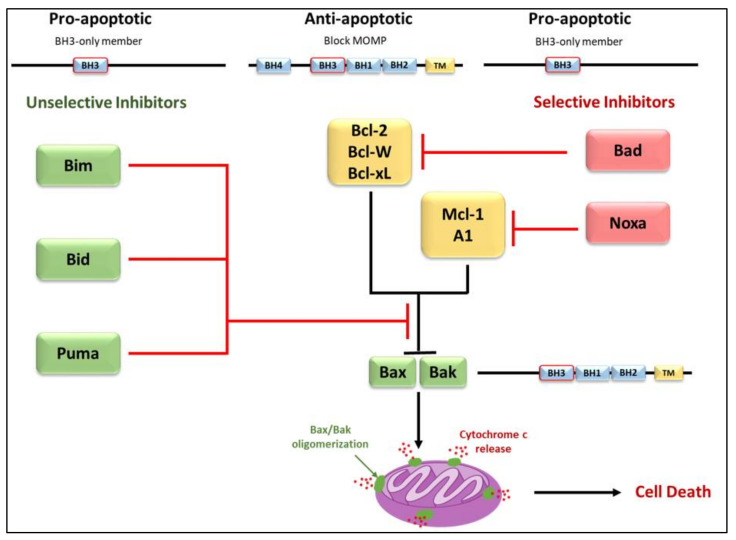
Differential binding of BH3-only proteins in the inhibition or progression of MOMP. To alleviate the inhibition that anti-apoptotic members have on Bax/Bak oligomerization, either a single unselective BH3-only member or multiple selective BH3-only members must bind. Selective members have an affinity for specific anti-apoptotic members whereas unselective members may bind to all members. Unselective members Bim, Bid, and Puma bind with high affinity and are considered potent inducers of apoptosis. Due to the selective nature of Bad and Noxa, these members are considered weak inducers.

The convergence of pro-apoptotic signal-transducing molecules or cytotoxic stimuli to the outer mitochondrial membrane induces MOMP, releasing cytochrome c into the cytosol and promoting apoptosome formation and caspase activation (Figure 4) [18,43,72,79,80]. MOMP is under the control of the Bcl-2 family of proteins. They are subdivided into three categories; pro-apoptotic proteins containing three Bcl-2 Homology (BH) domains, pro-apoptotic BH3- only proteins lacking BH domains 1 and 2, and anti-apoptotic proteins [1,43,72,79,81]. The anti-apoptotic Bcl-2 family proteins; Mcl-1, Bcl-2, Bcl-xL, Bcl-w, and A1, function to block MOMP. Overexpression of these proteins, which commonly occurs in cancer, inhibits stress-induced apoptosis [18,79]. The pro-apoptotic BH-1,2,3 proteins; Bax, Bak, and Bok, share three homology domains such as those of anti-apoptotic Bcl-2 family members and are considered essential for the progression of the cellular death pathway [12,79]. Finally, the pro-apoptotic BH3- only proteins; Bid, Bim, Bad, Noxa, and Puma do not have BH domains 1 and 2, possessing only the BH3 domain [79,81]. Interactions amongst the Bcl-2 proteins regulate MOMP [1,79,81]. Suppression of Bax/Bak activation by the antiapoptotic Bcl-2 family members can be overcome by the pro-apoptotic BH3-only proteins (Figure 4). These proteins act as stress sensors and are regulated both transcriptionally and through post-translational modifications [47,82].

### 3.3. Elucidation of the Caspases Reaction Mechanism

Caspases stand for Cysteine (Cys)-dependent aspartate-directed proteases are a sub-branch of the enzymes family proteases which possess an indispensable role in apoptosis, as mentioned previously. In a general description, caspases work on the cleavage of protein by activating the nucleophilic site of Cys [83]. Understanding the chemical reactivity of caspases is considered, with no doubts, the main trigger of developing drugs capable of enhancing or blocking apoptosis in case of any disturbance in this balance. For the latter reasons, several authors were interested in the elucidation of the working mechanism of caspases during cell death [84,85,86,87,88,89,90,91].

**Figure 5:**
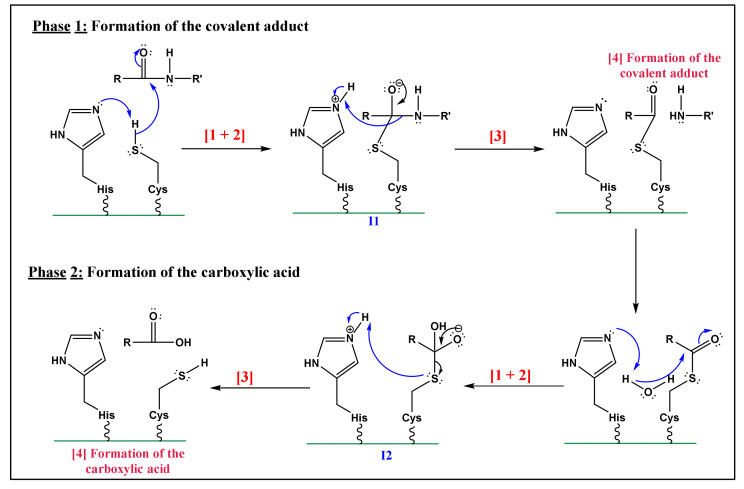
The assumed reaction mechanism for Cys protease. (Adapted with permission from Miscione et al. ref. [83]. Copyright 2010 American Chemical Society). (Numbers on the arrows are added to refer the reader where each step is exactly explained in the text).

The commonly proposed mechanism is described in Figure 5 and is divided into two principal parts. It describes the cleavage of the peptide bond (amide function). The below steps can be recognized:

For phase 1: Formation of the covalent adduct

1. Nucleophilic activation: the alkaline property of one of the nitrogen atoms, in the imidazolic part of Histidine (His), deprotonates the hydrogen in the thiol (-SH) group of the Cys residue, yielding a thiolate.
2. Thiolate nucleophilic attack on carbonyl: the carbonyl group of the aspartic peptide bond undergoes a nucleophilic attack by the yielded thiolate in the latter step. This contributes to the formation of a first tetrahedral intermediate (I1; Figure 5).
3. α-amino protonation: the amine group of I1 constitutes a good leaving group. This will enhance the possibility of the protonation of the α-amino moiety by the previous protonated nitrogen of the His residue.
4. Formation of the covalent adduct: the acyl-enzyme complex and the cleavage of the peptide bond.

For phase 2: Hydrolysis of the covalent adduct

The catalytic cycle completion yields the formation of carboxylic acid from the starting peptide:

1. Once, again the alkaline property of one of the nitrogen atoms in the imidazolic part of His deprotonates a water molecule.
2. This deprotonation contributes to the formation of a hydroxide. The strong alkaline and nucleophilic property of the hydroxide contributes to the attack of the electrophilic site of the carbonyl function. This yields a second tetrahedral intermediate (I2; Figure 5).
3. α-thio protonation: Similarly, to the third step of phase 1, the sulfur in I2 constitutes a good leaving group. This enhances the possibility of protonation of the *α-thio* moiety by the previously protonated nitrogen of the His residue.
4. Formation of the carboxylic acid by regeneration of His and Cys counterparts.

The proposed mechanism is a straightforward application of Cys and His reactive sites. Yet it had been not proved by any experimental and/or theoretical studies [89]. Hence, some important features of the mechanism can be found in Figure 5 are rather unclear. First, the large distance between the alkaline nitrogen of His and the hydrogen of Cys (6–7 Å) makes the first protonation more likely unfortunate [85,89,90]. Another assumption states that the two active sites exist as ion-pair by a full transfer of electrons from Cys hydrogen to His nitrogen [86,87,88] ruled out the ionic bonding model assumption with molecular dynamics calculations. As a result, they suggested that the lack of a proton acceptor in the water simulation could have a significant impact on the reaction’s pathway. Furthermore, it is reliable to consider that the catalytic site of Cys is not previously polarized, and that protonation of His nitrogen occurs during the reaction [84,87,88,89]. This statement goes along with pH values of caspases lying between 6.8 and 7.4 [83].

Second, the protonated nitrogen is not in a good position to protonate the α-amino group of I1 [84], as referred to in Figure 5 (phase 1). Brady et al. [84] proposed that a molecule of water interacts with Gly-145 (case of caspase-7) and that water acts as a donor of hydrogen due to its protic behavior. Additionally, these authors highlighted the function of His as a stabilizer of the charge developed on the leaving group by forming an ion pair. Once protonated, this residue has the capacity to interact with the oxygen of the thioemiketal (I1; Figure 5), after the nucleophilic activation (step 1 of phase 1; Figure 5). This will yield to the formation of a covalent bond between the carbon and Cys [92] (Figure 5).

**Figure 6:**
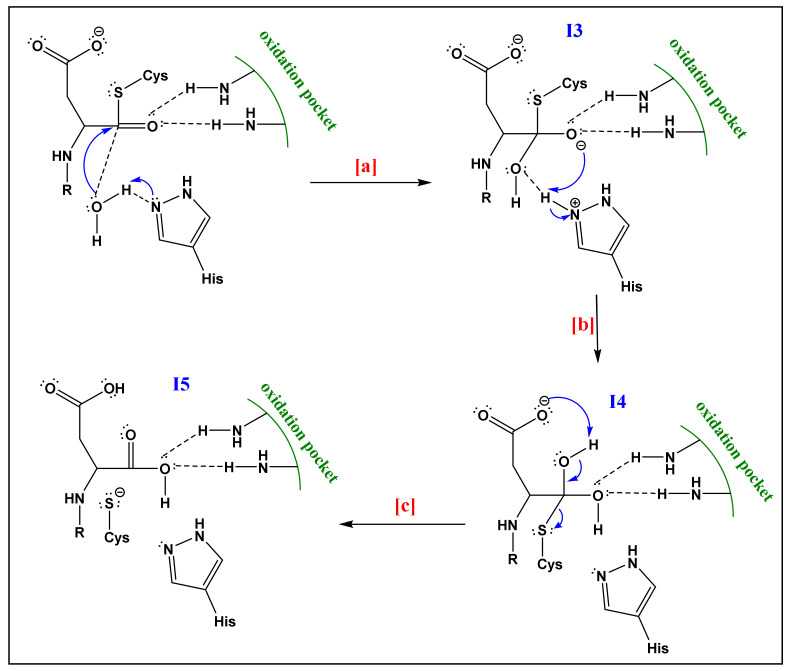
Mechanism of caspase-3 as proposed by Sulpizi et al. [87]. (Adapted with permission from Miscione et al. ref. [83]. Copyright 2010 American Chemical Society). (Letters on the arrows are added to refer the reader where each step is exactly explained in the text).

For all up-mentioned reasons and assumptions, Sulpizi et al. [88] proposed another mechanism and performed a Quantum Mechanics/Molecular Mechanics simulation to examine in detail the mechanism of caspase-3, focusing their attention on the hydrolysis of the covalent adduct phase (Phase 2; Figure 5). The proposed and simulated mechanism by these authors involves two water molecules, working as proton donors. The first water molecule worked on the formation of a hydrogen bond with the His. This interaction favors the alkaline/nucleophilic attack of water to the carbonyl function of Cys. The second one enters the active site from the solvent bulk during the molecular dynamic simulation. The results of this theoretical study, which discarded the previously postulated mechanism (Figure 5) is summarized as follows (see Figure 6):

- The already formed hydrogen bond between His and the first water molecule will favor the deprotonation of the latter. The yielded hydroxide attacks the acyl-enzyme complex on the carbonyl moiety.
- The yielded alkoxide in I3 will attack the proton already captured by the His part in (a). This will form a germinal diol (I4; Figure 6).
- The carboxylate function of the side-chain aspartate will attack one of the hydrogens of the diol. The thiol, acting as a good leaving group, will enhance the possibility of the formation of a carbonyl bond; thereby a carboxylic acid and a thiolate (I5; Figure 6). The computational investigation shows that for the attack of the water molecule, a free energy barrier of about 19 ± 4 kcal/mol must be overcome, these trends are following the experimental results of Sulpizi et al. [88].

In another study, Miscione et al. [83] focused on the first phase of the mechanism. The authors performed a Density Functional Theory (DFT) computational study in the mechanism of caspase-7. DFT determines the electron density of a molecule, thereby deriving properties and reactivity of the molecule. One of the main advantages of DFT is its capacity to increase computational accuracy without any additional increase in computing time [91]. Additionally, DFT can give detailed and important information on the restricted spot such as the active sites of the enzyme [83].

**Figure 7:**
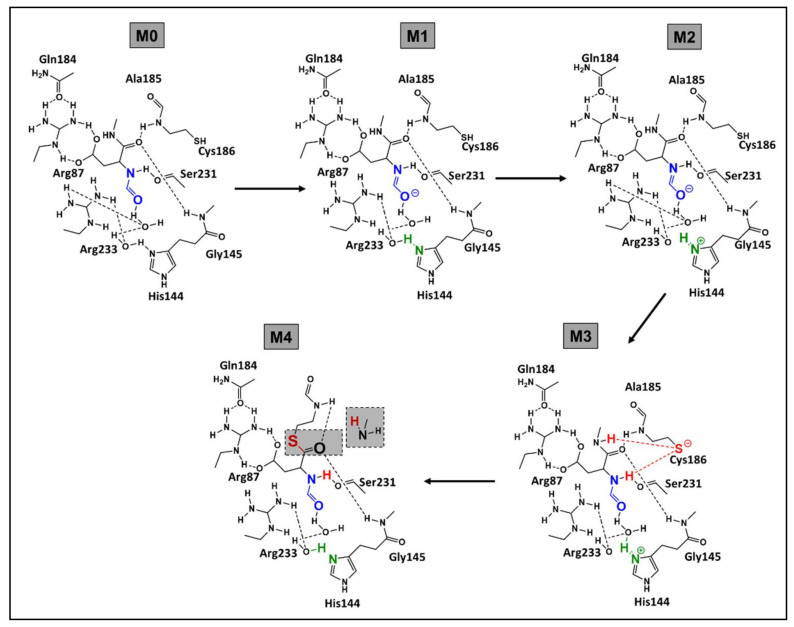
Mechanism of caspase-7 as proposed by Miscione et al. (Adapted with permission from Miscione et al. ref. [83]. Copyright 2010 American Chemical Society) (Colors are assigned to let the reader have a better follow-up of the explanation in the text).

Following their calculations, Miscione et al. [83] have revealed the existence of a novel mechanism that involves the activation of a catalytic dyad of His and Cys. The conventionally proposed path cannot take place because of the large distance between His’s nitrogen and Cys’s hydrogen. This will exclude any possibility of a direct proton transfer between the dyad. The proposed mechanism of Miscione et al. [83] is composed of three kinetic steps (relative to the formation of M1, M2, and M3; Figure 7).

The first two steps consist of the protonation of the alkaline nitrogen of His-144 by a water molecule (highlighted in green; Figure 7). In the third step, Cys-186 is activated by deprotonation of the thiol, yielding the thiolate which is a strong nucleophile (highlighted in red; Figure 7). The final step, yielding to the final product, consists of a complex proton transfer where the aspartate (carboxylate function) and one water molecule capture protons. The protonated His residue “assists” the breaking of the peptide bond by protonating the leaving group. The protonation involves a water molecule that again behaves as a proton shuttle. This process is made possible by the favorable arrangement reached by the His N-H bond, a water molecule, and the –NHMe substrate group (simulating the leaving group) after Cys activation. In brief, to overcome the large distance between Cys and His active sites, Miscione et al. [83] relied on the role of water molecules to act as a mediator of proton transfers. These assumptions were supported by theoretical calculations that gave energy barriers in the acceptable range.

In another part, Miscione et al. [83] proposed an alternative mechanistic path that consists of one kinetic step. This mechanism yields bond cleavage directly from M0 and the role of His in the deprotonation of Cys is discarded. Yet this mechanism required high geometrical distortions contributing to a high energy barrier (32.2 kcal/mol) for a one-step reaction channel [83]. Neglecting the possibility of a one-step reaction confirms the relevance of the catalytic dyad (His and Cys) in the peptide bond cleavage.

## 4. BH3-Only Protein Noxa

### 4.1. Discovery

In 1990, Noxa was first identified as a cDNA clone during a screen in adult T-cell leukemia cells for gene products involved in tumorigenesis by Hijikata et al. [93]. When peripheral blood mononuclear, human embryonic lung, and Jurkat T acute lymphoblastic leukemia cells were treated with the tumor promoter mitogen phorbol-12-myristate-13-acetate (PMA) Hijikata et al. [93] observed a rapid induction of a novel transcript, which he named ATL-derived PMA-responsive gene. Under the HUGO system, it was later termed PMA-induced protein 1 (PMAIP1). [94]. In a separate study, a cDNA was identified in X-ray-irradiated wild-type and *IRF-1/p53* double deficient mouse embryonic fibroblasts (MEF) [95]. This cDNA was termed *Noxa* (Latin for damage), and a human homolog of *Noxa* was identified in Saos2 cells [95]. Though both PMAIP1 and Noxa are both acceptable names for this protein, many current studies, including utilize Noxa for simplicity.

### 4.2. General Features and Transcript Variants

Human Noxa encodes a 54-amino acid protein that contains a single Bcl-2 homology 3 (BH3) domain [95] and a C-terminal MTD (stands for mitochondrial targeting domain) [96] that are both conserved between multiple mammalian species. The core gene structure of human Noxa contains three exons and two introns [97]. To date, three splice variants of human Noxa have been identified. Transcript 1, which includes exon 1 and 3, encodes for the 54 amino acid Noxa where both the BH3 domain and MTD are encoded within exon 3.

Transcripts 2 and 3, named Noxa-splicing variants 1 and 2, respectively (*NSV-1/2*), both contain exons 1 and 3, although *NSV-1* contains a portion of exon 2 (2a), and *NSV-2* contains the entire exon 2 [97]. Sequence analysis of *NSV-1* and *NSV-2* predict the synthesis of 136 amino acid and 70 amino acid proteins respectively [97]. Both *NSV-1/2* lack BH3 domains, due to differences in the reading frame of *NSV-1/2* as compared to Noxa, and have extremely short protein half-lives, as they are undetectable without treatment with the proteasome inhibitor MG132 [97]. The in vivo function of these Noxa splice variants is still to be determined.

### 4.3. Regulation of Noxa Expression and Post-Translational Modification

Early observations indicated that Noxa transcription was primarily induced by p53. In wild-type and IRF-1-deficient MEFs, X-ray irradiation caused rapid induction of Noxa mRNA, whereas no Noxa induction was observed in p53¯/¯ MEFs [95]. Analysis of the Noxa promoter region revealed a bona fide p53-response element 195 bp upstream of the transcription start site [95,96]. Additional studies have investigated p53-dependent transcription of Noxa either by in situ hybridization in p53¯/¯ mice or treatment of multiple cell lines with various chemical compounds [98,99,100,101]. The p53 independent regulation of Noxa by various stimuli has also been observed. Noxa induction was observed when multiple p53¯/¯ melanoma cell lines, along with PC-3 prostate cells and Saos-2 osteosarcoma cells (both p53 null cell lines) were treated with the γ-secretase inhibitor GSI [102]. Hypoxia-induced HIF-1α has been shown to induce Noxa mRNA and protein expression in H719 and Saos-2 cells independent of p53 by binding to a hypoxia response element (HRE), at −1275 bp, within the Noxa promoter [60]. Overexpression of adenovirus E1A protein, in the neuroblastoma cell line SH-SY5Y (non-functional p53) and SaOS-2 cells, results in activation of p73 and induction of Noxa mRNA [103]. H_2_O_2_-induces activating transcription factor 4 can induce Noxa mRNA expression in Jurkat cells by binding to a cAMP response element-binding site within the Noxa promoter [104]. A FoxO-binding site has been identified within the Noxa promoter by treatment of Jurkat cells with α-tocopheryl succinate, resulting in activation of FoxO1 and FoxO1-mediated transcription of Noxa [105]. Additional studies have been completed that have investigated p53-independent regulation of Noxa transcription [106,107,108,109,110,111,112].

In addition to transcriptional regulation of Noxa, proteasomal degradation has been implicated in the control of Noxa protein stability. Noxa has a short half-life [97], although it does not contain any PEST or known E3-ligase binding domains. KLF6-SV1 was observed to bind to Noxa and lead to its HDM2-mediated proteasomal degradation upon KLF6-SV1 overexpression in SKOV3 cells [113]. Additionally, proteasome inhibition in SKOV3 cells by MG132 causes an increase in both KLF6-SV1 and Noxa [113]. Treatment of MM.1S cells with the novel proteasome inhibitor MLN2238 results in increased expression of both p53 and Noxa [114]. Treatment with the proteasome inhibitor bortezomib (PS-441, Velcade) results in Noxa mRNA and protein induction in both p53 wild-type and p53-null melanoma cells, but not in normal melanocytes [115]. This effect of bortezomib was also observed in vivo and multiple other cell lines [116]. A cellular myelocytomatosis viral oncogene (c-MYC) binding site within the Noxa promoter has been identified, and siRNA knockdown of c-MYC reduced bortezomib-induced Noxa mRNA expression in multiple melanoma cell lines, MDA-MB-231 cells, and HeLa cells [117,118]. Treatment of LX-2 cells (human hepatic stellate cells) with MG132 resulted in increased expression of both Noxa mRNA and Noxa protein, and the MG132-induced apoptosis in LX-2 cells was shown to require Noxa [119]. Recently, siRNA knockdown of the E3 ligase, Sensitive to Apoptosis Gene (SAG) was found to lead to an increase in Noxa, whereas overexpression of SAG leads to a decrease in Noxa [120]. However, further studies into the interaction between SAG and Noxa are needed, as it has not been identified if SAG interacts directly with Noxa or if Noxa is ubiquitinated by SAG.

Post-translational modifications, such as ubiquitination and phosphorylation, have been shown to have essential roles in the function and degradation of many different proteins. Baou et al. [121], demonstrated that ubiquitinated Noxa can be detected in both untreated and MG132-treated HEK293T cells when transfected to overexpress HA-tagged or untagged Noxa, leading to proteasome-dependent degradation of Noxa [121]. To date, a specific E3 ligase responsible for the ubiquitination of Noxa has not been identified. It has been demonstrated that Noxa is phosphorylated on Ser13 by the atypical cyclin-dependent kinase CDK5 in the presence of glucose [122] and that phosphorylation of Ser13 prevents the pro-apoptotic function of Noxa [123]. Furthermore, immunoprecipitation of Mcl-1 revealed that Mcl-1 is unable to associate with phosphorylated Noxa, although analysis of phospho-Noxa/Mcl-1 interaction was measured using a phospho-Ser13-Noxa mouse monoclonal antibody that was shown to detect phospho-Noxa generated from in vitro kinase assays or in vivo only when Noxa was ectopically overexpressed [123]. No data has been published to date on whether phosphorylation of Noxa on Ser13 affects Noxa degradation or whether endogenous Noxa is phosphorylated in vivo by CDK5 [65].

Heat-shock is another cellular stress that has effects on apoptosis. A 2.95-fold up-regulation of Noxa mRNA expression was observed in one study that investigated the effects of heat-shock on IMC-3 cells [124]. In a separate study, it was observed that heat shock causes Noxa protein levels to initially drop, but then to return to higher-than-basal levels in PErTA cells, a human acute lymphoblastic T cell line derived from the PEER cell line that expresses rtTA [82]. Additionally, the depletion of Noxa by shRNA prevented heat-induced apoptosis in PErTA cells [82]. The mechanism of heat-induced Noxa expression is currently unknown.

### 4.4. Subcellular Localization and Association with Bcl-2-like Proteins

Assessment by immunostaining has shown that overexpressed mouse Noxa preferentially localizes to the mitochondria and that mutations within either the BH3 domain or MTD prevent mitochondrial localization [95,96]. Furthermore, Noxa constructs that are missing either the BH3 domain or MTD fail to induce apoptosis [96]. This suggests that both the BH3 domain and MTD are required for Noxa-induced apoptosis due to the proximity of the BH3 domain and MTD mutations in either domain may change the overall conformation of Noxa and impair Mcl-1 binding [94]. One study has also observed Noxa localization in the ER in melanoma cells where Noxa overexpression leads to the accumulation of intracellular Ca^2+^ [125]. The mechanism behind Noxa-induced Ca^2+^ accumulation is still unknown.

Noxa contains only a single BH3 domain, placing it in the growing category of pro-apoptotic BH3-only proteins. Studies have shown that overexpression of Noxa can significantly induces apoptosis in various cell lines [95,96,116], as well as correlates with MOMP, reactive oxygen species (ROS) accumulation, and cytochrome *c* release [92,96,125]. Additionally, *Noxa*¯/¯ mice show a severe osteoporotic phenotype due to the increased survival of short-lived osteoclasts [126]. Conversely, other studies have shown that Noxa overexpression has little to no apoptotic potential [66,127,128], and that Noxa knockdown has no developmental effect in mice [129,130]. Additionally, mature neutrophils, differentiated from established neutrophil progenitor cells from Noxa-deficient mice, have a slight resistance to spontaneous apoptosis [131]. This result was also observed in cultured primary neutrophils from Noxa-deficient mice, though substantially reduced apoptosis was observed in cultured primary neutrophils and mature neutrophils both established from Bim/Noxa-deficient mice [131]. Recently, Muenchow and co-workers used the CRISPR/Cas9 knock-out and verify that both Bax and Noxa are crucial for ABT-199/BZB-induced apoptosis. Additionally, they proved that combined inhibition of Bcl-2 and Noxa are involved for cell death induction by ABT-199/BZB [132].

**Figure 8:**
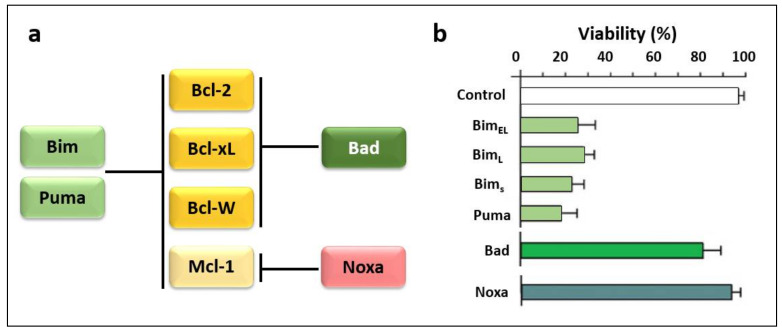
Schematic illustration (**a**) and viability assays in fibroblasts results (**b**) depict the selective interaction of Noxa with anti-apoptotic Bcl-2 family proteins (Adapted with permission from Chen et al. ref. [67]. Copyright 2005 Elsevier).

One study observed that Noxa selectively binds to the pro-survival proteins Mcl-1 and A1, and that overexpression of Noxa alone has weak apoptotic potential in MEFs [67]. Figure 8 illustrates the selective interaction of Noxa with anti-apoptotic Bcl-2 family proteins [67]. As shown in Figure 8a, promiscuous members (Bim, Puma, tBid) can bind to all anti-apoptotic Bcl-2 family proteins, while selective members (Bad, Noxa) are able to bind only certain anti-apoptotic Bcl-2 family proteins [67]. Viability assays in fibroblasts (Figure 8b) demonstrated that certain BH3-only proteins (Bim_EL_, Puma) are potent killers when overexpressed, while others (Bad, Noxa) are weak killers. Conversely, a Noxa construct that contains the Bad BH3 domain instead of the Noxa BH3 domain proved to be a potent killer [67,133,134].

The specificity of Noxa towards Mcl-1 and A1 is dependent on key amino acid residues within the Noxa BH3 domain. Mutations in the Noxa BH3 domain (m3) allow Noxa to bind to Bcl-xL with a 100-fold increased affinity versus wild-type Noxa and is a more potent inducer of apoptosis [67], while other mutations within the BH3 domain rendered Noxa inactive [95,96]. Additionally, Bims chimeras containing the Noxa BH3 domain showed reduced apoptotic ability, as compared to the wild-type Bims, which have a high apoptotic potential. Bims-Noxa BH3 chimeras were also restricted to binding Mcl-1, further demonstrating how the Noxa BH3 domain controls binding specificity and apoptotic potential of Noxa [67]. Interaction of Noxa with Mcl-1 has also been observed in melanoma cells treated with bortezomib [135], and in MDN and Jurkat cells where endogenous Noxa/Mcl-1 complexes were detected [106,136]. A recent study observed that Noxa can bind to Mcl-1 and Bcl-xL in NB15-BclcL neuroblastoma cells demonstrating that Noxa can bind to different Bcl-2-like proteins in different cell types [128]. Additionally, treatment of HeLa cells with either UV, thapsigargin (ER stress inducer), or MG132 results in generation of Mcl-1-free Noxa which is then able to bind to Bcl-xL and induce apoptosis [136,137,138]. Another study has demonstrated that Noxa can bind to Bcl-2, though this interaction can only be observed in Jurkat cells when they are treated with either bortezomib or MG132. Conversely, it was also demonstrated that interaction between Bcl-2 and Noxa can be observed in vivo in RL cells (diffuse large B cell lymphoma) that constitutively overexpressed Bcl-2 and in human colorectal carcinoma cell line initiated from an adult male (HCT116) colon cancer cells that were treated with the topoisomerase I poison camptothecin, which causes upregulation of Noxa levels [139].

Due to the specificity of Noxa for both Mcl-1 and A1 [67], the cellular levels of Mcl-1 and A1 control sensitivity to Noxa-induced apoptosis. Overexpression of Noxa in MEFs leads to Mcl-1 degradation without significant induction of apoptosis [66]. Consistent with the idea that Noxa needs to be complemented by Bad, which targets Bcl-xL, Bcl-2, and Bcl-w, to induce apoptosis [67], overexpression of both Noxa and Bad was shown to induce apoptosis in MEFs [66,133]. Overexpression of Noxa in Bcl-xL¯/¯ MEFs induced Bak-dependent apoptosis, demonstrating that Mcl-1 and Bcl-xL constrain Bak and that Noxa specifically engages Mcl-1 to promote Bak-dependent apoptosis [66]. Overexpression of Noxa has also been shown to disrupt Mcl-1/Bak complexes in multiple myeloma and B-cell lymphomas and in Jurkat cells [140]. Noxa has also been shown to disrupt Mcl-1/Bim complexes in bortezomib-treated MDN cells [136].

Another feature of Noxa is the interaction between Noxa and Mcl-1 promotes proteasome degradation of Mcl-1. Willis et al. [66] demonstrated that overexpression of Noxa leads to proteasome-dependent Mcl-1 degradation. Furthermore, they showed that this Noxa-induced Mcl-1 degradation requires the association of Noxa with Mcl-1 [66]. Noxa-induced Mcl-1 degradation has also been observed when Noxa was overexpressed in U266 myeloma cells [140] and MEFs [38]. Mcl-1 basal levels appear to be modulated by the HECT- and BH3domain-containing Mule/ARF-BP1 E3 ligase [38]. Recently it has been demonstrated that overexpression of Noxa causes decreased Mcl-1/USP9X interaction and, conversely, increased Mcl-1/Mule interaction, overall leading to increased Mcl-1 ubiquitination and degradation [141]. Structural analysis of recombinant Noxa/Mcl-1 complexes has demonstrated that a C-terminal portion of the Noxa BH3 domain (FRQKLL) is required for Mcl-1/Noxa degradation [38]. Overall, these studies have established a pro-apoptotic role for the BH3-only protein Noxa through its interaction with Mcl-1.

## 5. BH3-Only Protein Puma

Puma, a p53 Up-regulated Modulator of apoptosis protein, was first discovered and cloned as a transcriptional target of p53 by two independent laboratories 19 years ago [142]. In the same year, Han and colleagues identified the bbc3 (Bcl-2 binding component 3) gene that corresponds to the Puma cDNA [143]. Puma is a highly efficient pro-apoptotic protein, thought to be one of the most powerful and effective “killers” among the BH3-only proteins. The bbc3 gene has been reported to encode 4 different forms (α, β, γ, and δ) of which only the α and β forms contain the BH3 domain and thus display the pro-apoptotic activity. The length of the α Puma transcript is 1.6–1.9 kb encoding a 193 amino acid protein [142]. This protein is highly conserved among vertebrate species, yet shows no significant homologies to any other known proteins aside from those with the BH3 domain [52]. It has been demonstrated in a localization study that Puma is mainly restricted to the OMM in humans [144]. The Puma gene is mapped to chromosome 19q13.3, a region that is frequently deleted in a large number of human cancers including B-cell malignancies, as well as neural, colorectal, and ovarian cancers [142]. When present, however, Puma can effectively trigger apoptosis and eliminate cancer cells in the span of only a few hours [37,134,144,145].

### 5.1. Regulation of the BH3-Only Protein Puma

Regulation of the Bcl-2 family occurs through distinct cytotoxic stimuli in a variety of ways, including enhanced transcription and post-translational modifications [146]. Importantly, Puma mRNA is induced by p53-dependent and p53-independent apoptotic stimuli in several cancer cell lines [147]. These results support the idea that the regulation of Puma mRNA levels and thus the pro-apoptotic activity of the protein represents a common target in different cell death pathways [52,143]. The complexity of Puma function results from this protein’s involvement with a vast number of physiological and pathological processes, including the immune response, cancer, and neurodegenerative diseases as well as bacterial and viral infections [11,145]. Regulation of Puma expression during programmed cell death is coordinated by different transcription factors, most notably p53 but also through the activity of several other transcription factors including p73, sp1, Fox03a, E2f1, CHOP, TRB3, AP-1, and c-Myc [52,145].

### 5.2. p53-Dependent Apoptosis

The Puma gene is a direct transcriptional target of the tumor suppressor p53 [54]. The mutual interaction between p53 and Puma is an efficient mechanism for preventing the growth and division of abnormal cells, thereby protecting against the development of cancer [148]. It is known that p53 is required for the induction of Puma in response to DNA damage, but can also act on Puma in response to oxidative stress, deficiency of growth factors, or viral infection [52]. More so, a lack of Puma expression is often associated with the mutation or deletion of p53 function, which contributes to over 50% of human cancers [71,149]. Furthermore, p53 acts as a sensor of cell stress, responsible for tumor growth inhibition by either cell cycle arrest followed by DNA repair or by causing apoptosis through activating the transcription of several pro-apoptotic genes, including Puma. p53-dependent regulation of pro-apoptotic Puma expression and subsequent apoptosis relies on the functioning of GSK-3 (Glycogen synthase kinase-3) and acetyltransferase Tip60, which control the choice between cell cycle arrest and apoptosis [150].

### 5.3. p53-Independent Apoptosis

Stimuli from stressed or damaged cells can up-regulate Puma expression either by p53-mediated activation or by other transcription factors [6]. Puma plays a very important role in p53-independent apoptosis involved in the removal of damaged cells during hypoxia, infection, and cytokine or growth factor depletion. These conditions are strong signals for apoptosis, which can lead to irreversible damage in cells and tissues [52,151]. During such pathological conditions, induction of Puma mRNA expression and activity level is due to the activity of other transcription factors, such as p73, Sp1, or Fox03a depending on the cell types [6,52,152]. Although the mechanism remains unknown, the regulation of Puma occurs mainly without the participation of p53 in compromised cells [153].

Both p53-dependent and p53-independent inductions of apoptosis via Puma are involved in the immune response after bacterial and viral infections [71,153,154]. The immune response starts with increased T cell proliferation but once the pathogen has been eliminated, the number of T cells needs to be controlled through apoptosis to decrease the immune response. Puma plays a role in T cell apoptosis and is driven both by p53 and Fox03a [52,155]. This ensures the proper functioning of the immune system to prevent pathological conditions, such as autoimmunity [155].

## 6. Ion Channels in Regulated Cell Death

**Figure 9:**
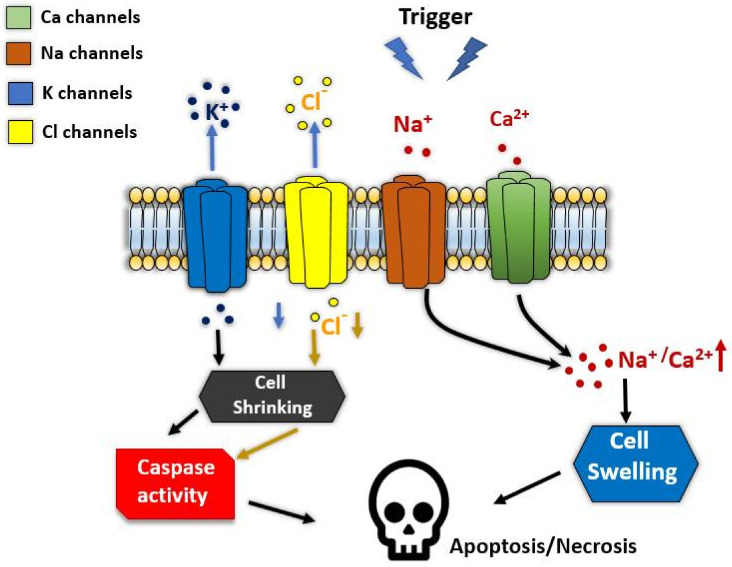
Ion channels involved in programmed cell death. The graphic diagram illustrates a simplified action of ion channels in apoptosis after being triggered by inflammation, ischemia, autoimmune disease, or cancer. Excessive activation of Ca^2+^ and Na^+^ channels leads to the accumulation of internal Ca^2+^ and Na^+^. As a result, the cell swells leading to necrosis. On the other hand, over activation of K^+^ and Cl¯ channels lead to massive efflux of these ions resulted in the reduction of K^+^ intracellular concentration induces caspases-dependent apoptosis. The efflux of Cl¯ is accompanied by efflux of water leading to shrinking and apoptotic cell death. Anionic channels can induce swelling by in-fluxing Cl¯ and free water as highlighted in the red dashed line. The figure was created with Biorender.com, accessed on 12 January 2022.

Ion channels are gated pore-forming TM proteins by interaction with ligands, or senses changes in voltage or mechanical stretch. These key membrane proteins mediate various cellular activities: starting from cellular communication, cell division to regulating metabolism and cell death. Ion channels participate in the execution of apoptosis, necroptosis, and caspase-independent cell death [156]. These different mechanisms of cell death require the transport of ions organic osmolytes and water altering the cell volume (by shrinking or swelling) that promote cell death (Figure 9).

Apoptosis is a genetically regulated process of cell death that is characterized by cytoplasmic and chromatin condensation, nuclear fragmentation, DNA-laddering, cytoplasmic blebbing, and the formation of apoptotic bodies [157,158,159]. Different forms of ion channels and pores have physiological roles in apoptosis via caspase-dependent pathways. For example, caspases-3, -6, and -7 activate mitochondrial permeability transition pore (MPTP), and Bcl-2 antagonist Bak leading to the loss of mitochondrial transmembrane potential and inhibit ATP production [160,161]. The Bax and Bak are multi-domain proteins sharing sequence homology within three to four Bcl-2 homology (BH) domains, including the main pro-apoptotic molecules, Noxa and Puma [162].

Calcium channels are known to be directly controlling cell volume, plasma membrane rigidity, and intracellular Ca^2+^ levels which could trigger apoptosis by a sustained Ca^2+^ increase [163,164,165]. Several Ca^2+^ releasing channels are localized in different organelles and cellular structures, including IP3 and ryanodine receptors in ER, and Ca^2+^ influx channels Orai1/Stim in the plasma membrane as well as the transient receptor potential channels TRPC1,3,6, TRPM2,7,8, TRPML2, TRPP5, and TRPV1,2,4 (for references see Lang and Hoffmann [166]; Figure 9). Besides, other TRP and cationic channels (such as purinergic P2X7, P2X1, Cav and Nav channels) are shown to be involved in apoptosis [161,167,168].

It is still unclear how the increase of internal Ca^2+^ and the followed shrinking of cells induce apoptosis. Nevertheless, Ca^2+^ activated potassium channels are having a key role in the process. However, the shrinkage is a consequence of results from a loss of K^+^ and cell water by activation of K^+^ channel [169,170]. Many types of K^+^ channels have been implicated in the activation of apoptosis, such as voltage-gated Kv channels, ATP-regulated K^+^ channels, two-pore K^+^ channels, and some types of Ca^2+^-dependent K^+^ channels (see Figure 9) [166,171,172]. Ion channels, as target for cancer apoptosis, are essential to maintain normal tissue homeostasis by controlling cell turnover. Imbalance in regulated cell death by inappropriate activation of ion channels may lead to neurological disorders, inflammation, and tissue reperfusion damage during a heart attack, graft rejection during transplantation, autoimmune disease, and cancer [156].

Potassium channels, including those to be identified, are shown to be upregulated in cancer cells that may be used as therapeutic targets to induce apoptosis in cancer cells [173,174,175]. The recruitment of signaling cascades by K^+^ channels may have been an additional impact on cell cycle progression and proliferation as non-conducting mechanisms of these channels [176]. Loss of cytosolic K^+^ due to activation of K^+^ channels is essential, but in some cell it is counteract apoptosis [166]. One particular Kv channel gained a specific interest in anticancer therapy is the Kv human ether à go-go 1 (hEag1, Kv10.1). It represents an interesting cancer target because of its ectopic expression in over 70% of human cancers [177]. In recent work, a novel bromotyrosine purpurealidin analog promoted cell death blocking Kv10.1 channel expressed in cancer and non-cancer cell lines [172]. The compound showed to be cytotoxic and appeared to induce apoptosis in all the evaluated cell lines by shifting the activation kinetics of Kv10.1 channel to more negative potentials. These results indicate the importance of developing selective modulators to Kv channels as leading anticancer drugs. Another candidate target for antiapoptotic drugs is Kv2.1 channel. This neuronal channel plays a prominent role in regulating the intracellular K^+^ which suppresses caspase function, inhibits the activity of nucleases, and limits the apoptosome formation [144,169]. After the ischemic, oxidative, or inflammatory insult, surface expression of phosphorylated forms of Kv2.1 channel would in turn permit K^+^ efflux in dying cells [178]. Designing a selective blocker for these mutated forms of Kv2.1 was shown to reduce ischemic stroke damage in the animal study, without adverse effect on Kv2.1 WT in healthy tissues, as a highly promising neuroprotective strategy [178].

Anion channels, predominantly chlorine channels (Figure 9), provides a mechanism maintain a relatively constant cell volume whenever an osmotic disequilibrium (increase in intracellular osmolytes or decrease in extracellular osmolality) favors the influx of free water [171]. Therefore, Cl¯ channels might be another target to induce apoptosis in cancer cells. Several forms of chlorine channels are shown to be mediating apoptosis, including volume-activated anion channel (VRAC), volume sensitive outwardly rectifying anion channel (VSOR), volume-sensitive organic osmolyte, and anion channel (VSOAC), The Cl¯ channel cystic fibrosis transmembrane conductance regulator (CFTR), and Ca^2+^-activating Cl¯ channels (Anoctamines [TMEM16 proteins]) which are reviewed by Kunzelmann, 2016. On one side, several activated Cl¯ channels induce apoptosis, including CIC-3 channels and anoctamins. Downregulation of VRAC channels was associated with multidrug resistance, which induces shrinkage and apoptosis [179,180] ANO6 is an example of an outwardly rectifying Cl¯ channel of this group [181]. On the other hand, ANO1 channel belongs to Cl¯ channels correlated with cell profanation and upregulated in tumor cells [182,183,184].

## 7. Heat Shock Response to Puma and Noxa Proteins Expression In Vitro

Puma expression is a strong inducer of apoptosis in stressed cells and therefore it is important to examine how stressful conditions such as heat-induced stress may affect Puma expression. With the expression of this vital regulator of apoptosis characterized, we can then go on to investigate what mechanisms are responsible for the altered expression of both Puma and Noxa seen in many disorders and diseases. These studies made use of the human acute lymphoblastic T cell line, PEER, that had been engineered to inducible overexpressing HSP70 (PErTA70) using the tetracycline-regulated expression system [185]. Our future work will focus on the effect of Puma protein expression in PErTA70 cells. Heat shock treatment of non-induced (− HSP70) and induced (+ HSP70) PErTA70 cells are expected to rapidly deplete of Puma protein in non-induced HSP70 cell lines [153]. We predict a minimal increase in Noxa protein levels compared to the basal levels during the time that the cells are undergoing apoptosis. In cells overexpressing HSP70, we predict Noxa levels to regain during incubation at 37 °C. Previous studies have shown that this heat shock treatment leads to apoptotic cell death in over 50% of the non-induced cells, whereas the induced HSP70-expressing cells are highly resistant [185]. HSP70 expression normally decreases the apoptotic response allowing resistance to the stress signal through its chaperone capabilities of refolding misfolded proteins and sending irreversibly damaged proteins to the proteasome for degradation [186]. The molecular mechanism preventing the expression of Puma protein following heat shock remains elusive, and could be the result of several regulatory processes including inhibition of transcription, mRNA degradation, or inhibition of translation. To assess the cause of this loss of Puma protein, Puma mRNA will be demonstrated under control and heat shock conditions in the following research paper. Noxa protein levels increased following heat shock treatment. This increase is due to the loss of miR-23a targeting Noxa mRNA and protein expression levels. miR-23a synthesis is repressed in hypothermic cells and cells HSP70 expressing cells resist this repression [187]. As a result, the protective role played by HSP70 from heat-induced apoptosis maintains miR-23a levels and thereby preventing Noxa protein accumulation.

An important consideration is that miRNAs cannot fully account for the 100% loss of both Noxa and Puma proteins in the stressed cells. Another explanation for this loss could be due to proteasomal/lysosomal degradation of these pro-apoptotic proteins that might be increased in cells exposed to induced stressed conditions such as hyperthermia. Consequently, despite the increased mRNA expression, which could be the result of increased transcription or altered mRNA stability, protein levels do not increase as the rate of its degradation is also enhanced. Many studies indicate that directly targeting the apoptotic machinery through the manipulation of miRNA expression may offer new hope for improved therapy for cancer and other autoimmune and infectious diseases.

## 8. Conclusions

By understanding and developing new in vitro tools to regulate the expression of the Bcl-2 proteins in stressed cells, we could potentially augment their function to stimulate the apoptotic response in tumor cells. We expect that a method to alter Noxa and Puma expression will lead to a novel pathway for treating aberrant apoptosis, which contributes to carcinogenesis and altered sensitivity to chemotherapeutic agents, in which expression of these pro-apoptotic proteins play an essential role. Our ongoing studies on stable cell lines expressing miRNAs and shRNA knockdowns will highlight their role in the heat-induced regulation of Noxa and Puma expression and other regulators of apoptosis that they have been implicated in controlling, in hopes of restoring protein expression under conditions of cellular stress.
